# Prenatal exposure to vitamin D from fortified margarine and risk of fractures in late childhood: period and cohort results from 222 000 subjects in the D-tect observational study

**DOI:** 10.1017/S000711451700071X

**Published:** 2017-04-10

**Authors:** Mina Nicole Händel, Peder Frederiksen, Clive Osmond, Cyrus Cooper, Bo Abrahamsen, Berit L. Heitmann

**Affiliations:** 1 Department of Clinical Research, Odense Patient Data Explorative Network (OPEN), Odense University Hospital, University of Southern Denmark, 5000 Odense C, Denmark; 2 Research Unit for Dietary Studies, Bispebjerg and Frederiksberg Hospital, The Parker Institute and the Institute of Preventive Medicine, 2000 Frederiksberg, Denmark; 3 Medical Research Council Lifecourse Epidemiology Unit, University of Southampton, Southampton SO16 6YD, UK; 4 Department of Medicine, Holbæk Hospital, DK-4300 Holbæk, Denmark; 5 Section for General Practice, Department of Public Health, Copenhagen University, Øster Farimagsgade 5, opg. Q, 1014, Copenhagen K, Denmark; 6 The Boden Institute, Charles Perkins Centre, University of Sydney, D17, Johns Hopkins Drive, Camperdown NSW 2006, Sydney, Australia; 7 National Institute of Public Health, University of Southern Denmark, Øster Farimagsgade 5A, 2. 1353 Copenhagen K, Denmark

**Keywords:** Epidemiology, Vitamin D, Fracture risk, Fortification, Children

## Abstract

Prenatal low vitamin D may have consequences for bone health. By means of a nationwide mandatory vitamin D fortification programme, we examined the risk of fractures among 10–18-year-old children from proximate birth cohorts born around the date of the termination of the programme. For all subjects born in Denmark during 1983–1988, civil registration numbers were linked to the Danish National Patient Registry for incident and recurrent fractures occurring at ages 10–18 years. Multiplicative Poisson models were used to examine the association between birth cohort and fracture rates. The variation in fracture rates across birth cohorts was analysed by fitting an age-cohort model to the data. We addressed the potential modification of the effect of vitamin D availability by season of birth. The risk of fractures was increased among both girls and boys who were born before the vitamin D fortification terminated in 1985 (rate ratio (RR) exposed *v*. non-exposed girls: 1·15 (95 % CI 1·11, 1·20); RR exposed *v*. non-exposed boys: 1·11 (95 % CI 1·07, 1·14). However, these associations no longer persisted after including the period effects. There was no interaction between season of birth and vitamin D availability in relation to fracture risk. The study did not provide evidence that prenatal exposure to extra vitamin D from a mandatory fortification programme of 1·25 µg vitamin D/100 g margarine was sufficient to influence the risk of fractures in late childhood, regardless of season of birth. Replication studies are needed.

Based on serum 25-hydroxy vitamin D_3_ (25(OH)D_3_) measurements, studies have shown a pronounced seasonal variation in vitamin D status in Denmark and other countries with latitudes above 35° North and South^(^ ^1^ ^,^ ^2^ ^)^, most likely related to insufficient actinic vitamin D synthesis during the darker part of the year in such countries. Consequently, the vitamin D status will then depend more on diet, supplementation and/or fortification alone. In Denmark, the reintroduction of fortified foods to improve the general population’s vitamin D status is currently being considered, and its justification is part of an ongoing international debate.

Until 1 June 1985, vitamin D fortification of margarine was mandatory in Denmark, but fortification was abolished, related to an unsupported assumption that the amounts added to margarine were too small to impact the dietary needs of vitamin D in the Danish population^(^ ^3^ ^)^. We used this historical change in fortification legislation to examine the influence of extra prenatal vitamin D exposure from fortified margarine on the risk of fractures during pubertal-related growth spurts, which has the highest fracture incidence^(^ ^4^ ^)^.

Vitamin D and its metabolites play essential roles in regulating Ca homoeostasis in the intestine, kidney and bone. The evidence for linking maternal vitamin D insufficiency to offspring fracture rates is sparse and is based on results from animal studies^(^ ^5^ ^)^, as well as observational studies suggesting that maternal vitamin D status influences fetal bone growth and mineralisation^(^ ^6^ ^)^, and perhaps also long-term bone health among offspring^(^ ^7^ ^–^ ^12^ ^)^. Given that low bone mineral density (BMD) is predictive of increased fracture risk, as shown in both case–control and prospective studies – though principally in adults^(^ ^13^ ^–^ ^20^ ^)^ – maternal vitamin D insufficiency may potentially also influence fracture risk.

Among children and adolescents, the most common fracture site is the forearm^(^ ^21^ ^–^ ^25^ ^)^, followed by the carpal bones, clavicle and foot/ankle^(^ ^26^ ^)^. Across European countries, the seasonality of fractures exhibit notable similarities, with peaks during summer, with a notable drop in the month of July and a nadir during winter^(^ ^4^ ^,^ ^22^ ^,^ ^24^ ^,^ ^25^ ^,^ ^27^ ^,^ ^28^ ^)^.

We hypothesised that individuals born during the last 2 years of the mandatory vitamin D fortification had a reduced risk of sustaining fractures of the forearm, wrist or scaphoid bone, clavicle and ankle in late childhood, compared with those born 2 years after the termination of vitamin D fortification, allowing for a washout period after termination. In addition, it was hypothesised that the vitamin D fortification during sun-deprived months of gestation would be associated with the greatest risk reduction of offspring childhood fractures.

## Methods

### Study design

The D-tect study design^(^ ^29^ ^)^ relies on a natural experiment, defined as an exposure to an event or intervention, which has not been manipulated by the researcher^(^ ^30^ ^)^. The D-tect study is based on the fact that until 1 June 1985 it was mandatory in Denmark to fortify all margarine with vitamin D. Margarine was fortified with 1·25 µg/100 g and approximately 13 % (3–29 %) of all dietary vitamin D is estimated to have come from the fortified margarine^(^ ^31^ ^)^.

We did not identify other abrupt societal changes during 1983–1988 that potentially could influence our results, neither in relation to fortification practices in other food products for consumption^(^ ^31^ ^,^ ^32^ ^)^, nor in relation to margarine intake in the Danish population^(^ ^33^ ^)^ or in relation to national recommendations for vitamin D supplementation to pregnant women or infants. Therefore, any confounding would be expected to have influenced the exposed and non-exposed individuals from the two groups similarly, making them fully comparable. Hence, prenatal exposure to extra vitamin D from fortification is assumed to be the only parameter that separates the individuals in the two exposure groups.

### Study population

**Fig. 1:**
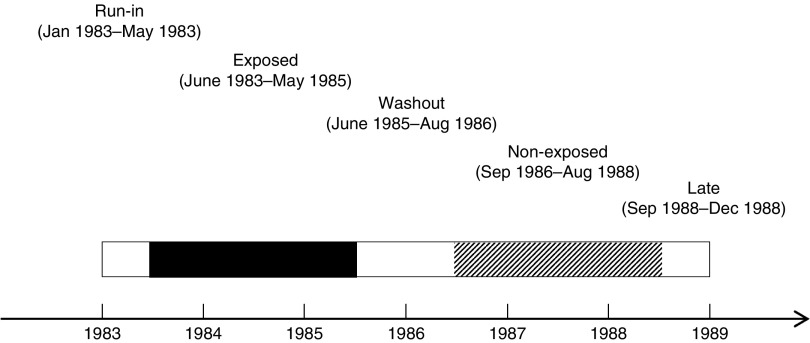
Definition of the exposure groups. Vertical lines indicate the timing of the cohorts around the vitamin D fortification termination date (31 May 1985). ■, Exposed; 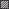, non-exposed; □, run-in, washout and late cohort.

All individuals born alive in Denmark from 1 January 1983 to 31 December 1988 were included in the study. We divided individuals from the birth cohort into different exposure groups. The exposed individuals were defined with birth dates from 1 June 1983 to 31 May 1985, and the non-exposed individuals were those with birth dates from 1 September 1986 to 31 August 1988. Between the exposed and the non-exposed groups, we included a washout period (9 months of pregnancy plus 6 months of margarine shelf-life) in the time period from 1 June 1985 to 31 August 1986. In order to use the full potential of the data set available, we included a run-in period with individuals born from 1 January 1983 to 31 May 1983, and a late period running from 1 September 1988 to 31 December 1988 (Fig. 1). Hence, individuals born before the termination of the mandatory fortification were exposed prenatally, but not during childhood (the exposed group), and those born after the fortification termination were neither exposed prenatally nor during childhood (the non-exposed group).

Follow-up time for fractures for each participant started at age 10 years (or the age at 1 January 1996 if the participant at that date was older than 10 years) and ended at death, emigration, disappearance or age 18 years, whichever came first. An overview of the study population is presented in Table 1.

**Table 1:** Number of individuals contributing with risk time, person-years, and number of fracture events in the study population by birth cohort exposure groups and sex

	Run-in	Exposed	Washout	Non-exposed	Late	Total
Boys (total)	10 974	53 555	35 308	58 542	9665	168 044
Person-years	54 035	321 870	263 149	446 284	74 230	1 159 568
Fractures (total)	1298	7636	5984	9623	1566	26 107
Forearm, wrist or scaphoid bone	950	5706	4521	7361	1221	19 759
Ankle	207	1101	830	1241	187	3566
Clavicle	141	829	633	1021	158	2782
Girls (total)	10 458	50 851	33 580	55 035	9286	159 210
Person-years	52 875	313 792	255 056	426 974	72 442	1 121 138
Fractures, total	673	4694	4063	6435	1051	16 916
Forearm, wrist or scaphoid bone	490	3609	3258	5108	878	13 343
Ankle	136	805	597	918	123	2579
Clavicle	47	280	208	409	50	994

Using the civil registration numbers, each individual was linked to the Danish National Patient Registry (NPR) for incident and recurrent diseases. The register contains information about hospital contacts, including diagnosis codes and procedure codes for all treatments at Danish hospitals^(^ ^34^ ^)^. The main study outcome was fracture of the forearm, wrist or scaphoid bone (International Classification of Diseases (ICD)-10: S52, S62·0); fracture of the clavicle (ICD-10: S42·0); and fracture of the ankle (ICD-10: S82·5, S82·6, S82·8). From 1994, ICD-10 diagnoses were classified according to the WHO International Classification of Diseases, and from 1 January 1995 the outpatient and emergency room contacts were mandatorily included in the registers^(^ ^35^ ^)^. Because of this incompleteness in the NPR, the individuals were considered at risk only from their age as on 1 January 1996 onwards. Recurrent fracture events were allowed, but if an individual had more than one fracture admission per event, we counted only the first admission and embedded a washout period of 6 months after a fracture at the same anatomical location, in order to avoid inflating the fracture risk estimates by including readmissions.

Risk time (person-years) and the number of fracture events were classified by sex, date of birth, age and calendar time during follow-up in monthly classes in a Lexis diagram^(^ ^36^ ^)^.

There are no ethical considerations regarding the epidemiological aspects of the study as only pre-existing databases and registries that had already been approved were used. According to Danish law, ethical approval is not required for purely register-based studies. Permission for conducting the study was granted by the Danish Data Protection Agency (J. no.: 2012-41-1156).

### Statistical analysis

Multiplicative Poisson models were used to examine the association between birth cohort and fracture rates. Rates were analysed by models for the number of fractures with the log-person-years as offset. With the Poisson model it is possible to explicitly model the underlying rates, and thus obtain estimates describing the fracture rates by age at occurrence, time of occurrence and time of birth (age, period, cohort). The analysis was performed for fractures overall and for fracture subtypes, separately for each sex. Power calculations for the present study have previously been published^(^ ^29^ ^)^. In brief, as the follow-up period varies, the final prevalence (=incidence×duration) was used, and it varied from 0·5 to 8 %. The significance level was *α* 0·05 and power *β* 0·80. The least detectable relative risk of outcome after change in fortification (assuming that either one (at least 100 000 individuals) or two birth cohorts are included) varied from 1·19 for a prevalence of 0·5 % to 1·05 for a prevalence of 8 %.

First, we described the variation in facture rates across birth cohorts by fitting an age-cohort model to the data. In a second series of models, the birth cohorts were divided into the five exposure groups mentioned above (run-in, exposed, washout, non-exposed, late period). In a third series of models, we addressed the potential modification of the effect of vitamin D availability by season of birth, with winter defined as births in November, December or January. We tested for interaction between season of birth and exposure in relation to fracture risk by likelihood ratio tests. In a fourth series of models, we included period effects. First, we fitted an age-period model to the data (with July 2001 as the reference period) and subsequently, a cohort-only model to the residuals (i.e. using the fitted values from the age-period model as offset). The estimated cohort effect is then the ratio between the observed number of fractures within the cohort and the expected number of fractures, where the expected number is based on the predicted rates from the age-period model. This sequential approach to age-period-cohort modelling corresponds to fixing the cohort effects to have no overall trend^(^ ^36^ ^)^.

All data management was performed in Stata version 13.1, and statistical analyses were performed in R (R Foundation for Statistical Computing).

## Results

In total, 327 254 children contributed with risk time during ages 10–18 years. We identified 104 406 individuals in the exposure period, of which approximately 51 % were boys, and 113 577 individuals in the non-exposed period, of which approximately 52 % were boys. In the run-in, washout and late periods, there were 21 432, 68 888 and 18 951 individuals, respectively. In total, 12 330 exposed and 16 058 non-exposed individuals sustained a fracture with an overall fracture rate of 19·4 (95 % CI 19·1, 19·7) and 18·4 (95 % CI 18·1, 18·7) per 1000 person-years among the exposed and non-exposed individuals, respectively. The fracture type with the highest incidence was forearm, wrist or scaphoid bone, with 9315 events in the exposed group and 12 469 events in the non-exposed group (Table 1).

### Fracture risk across age groups and seasonality in fracture occurrence

**Fig. 2:**
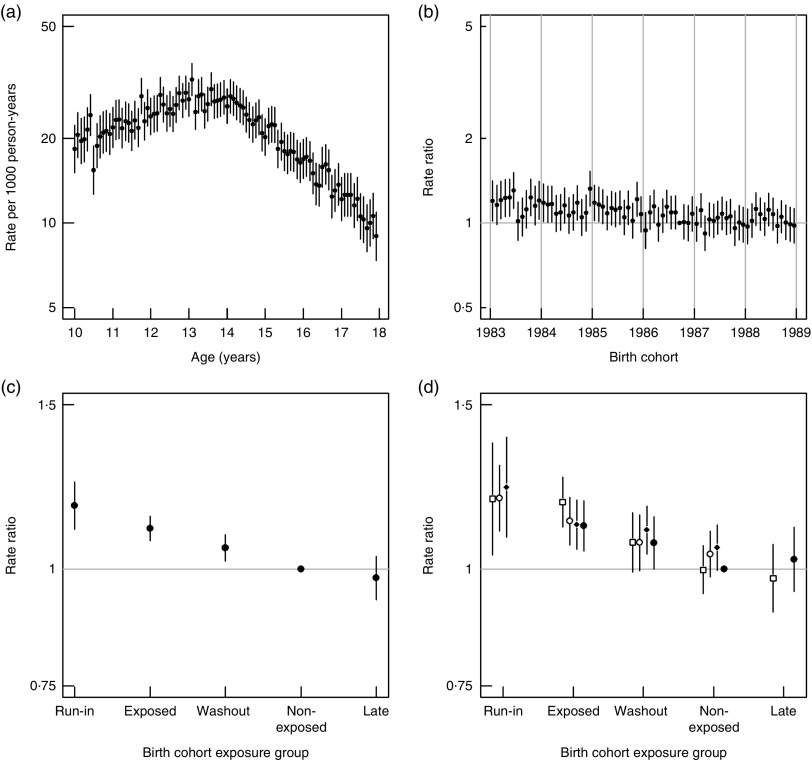
Age and birth cohort effects for boys born in 1983–1988. (a) Age specific fracture rates per 1000 person-years and 95 % CI for boys born in September 1986, (b) rate ratio relative to September 1986 cohort, (c) rate ratios by birth cohort exposure groups (‘Non-exposed’ cohort is reference) and (d) rate ratios by birth cohort exposure group and season of birth (birth season ‘August–October’ and ‘Non-exposed’ cohort is reference). 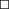, November–January; 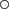, February–April; 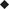, May–July; 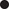, August–October.

**Fig. 3:**
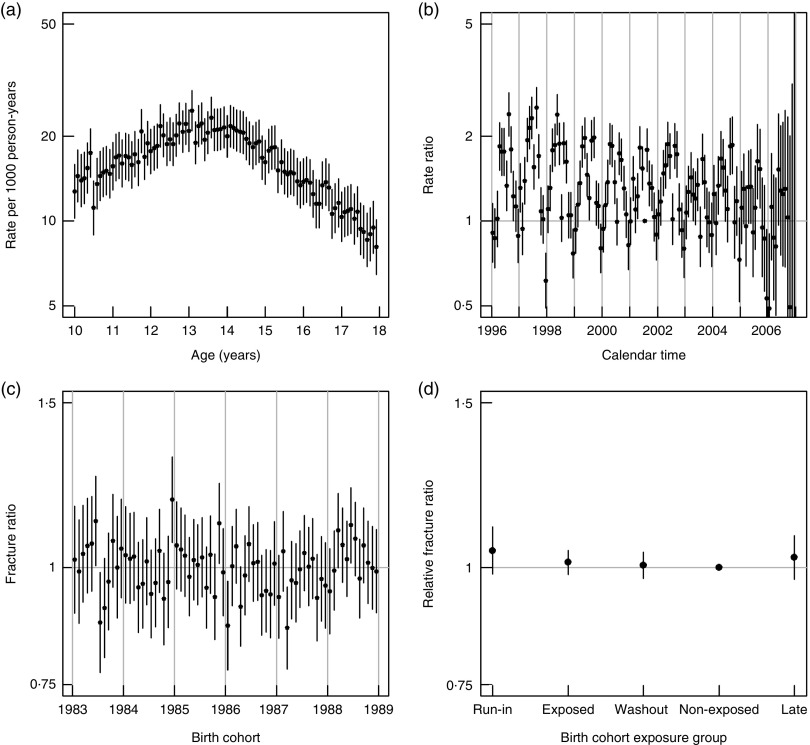
Age and period effects for boys with fractures occurring from 1996–2007. (a) Age specific fracture rates per 1000 person-years and 95 % CI for boys in June 2001, (b) rate relative to the July 2001 rate, (c) observed *v*. expected number of fractures conditional on the estimated age and period rates and (d) cohort effect by birth cohort exposure group relative to the cohort effect in the ‘Non-exposed’ cohort.

**Fig. 4:**
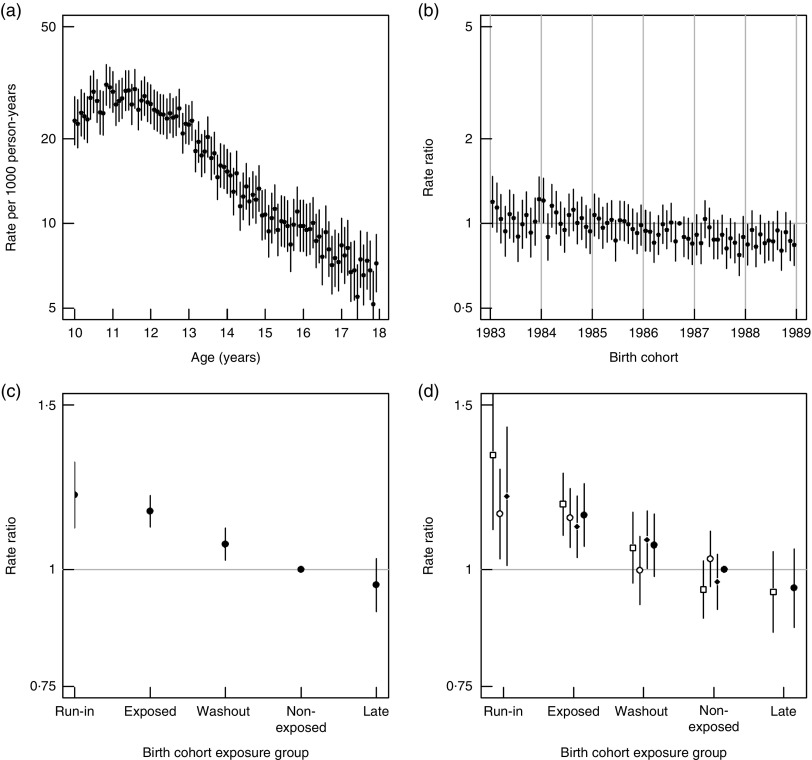
Age and birth cohort effects girls born in 1983–1988. (a) Age specific fracture rates per 1000 person-years and 95 % CI for girls born in September 1986, (b) rate ratio relative to September 1986 cohort, (c) rate ratios by birth cohort exposure groups (‘Non-exposed’ cohort is reference). and (d) rate ratios by birth cohort exposure group and season of birth (birth season ‘August–October’ and ‘Non-exposed’ cohort is reference). □, November–January; 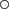, February–April; 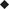, May–July; 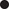, August–October.

**Fig. 5:**
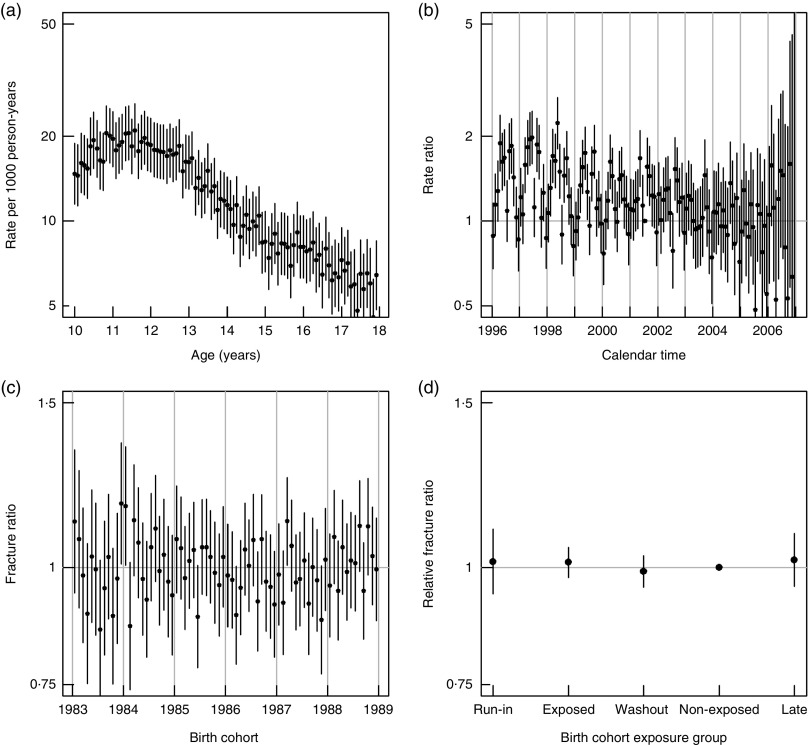
Age and period effects for girls with fractures occurring from 1996–2007. (a) Age specific fracture rates per 1000 person-years and 95 % CI for girls in June 2001, (b) rate relative to the July 2001 rate, (c) observed *v*. expected number of fractures conditional on the estimated age and period rates and (d) cohort effect by birth cohort exposure group relative to the cohort effect in the ‘Non-exposed’ cohort.

As expected, the fracture rates differed according to age and there was a rate difference between boys and girls. Among the boys, the overall average fracture rate was 22·5/1000 person-years, with a peak fracture rate of 35·1/1000 person-years between the ages of 13 and 14 years (Fig. 2 and 3). Among the girls, the overall average fracture rate was 14·6/1000 person-years, with a peak fracture rate of 27·9/1000 person-years between the ages of 11 and 12 years (Fig. 4 and 5).

The peak age of the ankle and clavicle fracture rates occurred later compared with the overall fracture rate, but was similar for both girls (range of 12–14 years) and boys (range of 15–16 years) (online Supplementary Fig. S1–S8). The overall average fracture rate was 2·7/1000 person-years for ankle and 1·7/1000 person-years for clavicle, respectively. Forearm, wrist or scaphoid bone fractures showed a similar pattern as the overall analysis, resulting from the high incidence rate of forearm, wrist or scaphoid bone fractures in children, which was the main contribution to the fracture outcome (online Supplementary Fig. S9–S12).

The estimates from the age-period model revealed a seasonal fracture pattern with increased risk during spring and early fall and with a nadir during winter in the period from 1996 to 2007 (Fig. 2 and 5).

A summary of the month-by-month periodicity is presented in Table 2. For both girls and boys, we observed an almost 2-fold increase in the fracture rate when comparing the months with the highest rates (April, May or August) and the month with the lowest rate (December).

**Table 2:** Age-adjusted facture rate by month of fracture (July is the reference) (Rate ratios (RR) and 95 % confidence intervals )

	Boys		Girls	
Month of fracture	RR	95 % CI	RR	95 % CI
January	0·92	0·86, 0·98	1·04	0·96, 1·13
February	1·04	0·97, 1·11	1·14	1·05, 1·24
March	1·21	1·13, 1·28	1·37	1·27, 1·48
April	1·48	1·40, 1·57	1·55	1·43, 1·67
May	1·67	1·58, 1·77	1·71	1·58, 1·84
June	1·44	1·36, 1·53	1·40	1·29, 1·51
July	1		1	
August	1·73	1·63, 1·83	1·57	1·45, 1·70
September	1·48	1·40, 1·57	1·65	1·53, 1·78
October	1·02	0·96, 1·09	1·20	1·11, 1·31
November	0·93	0·87, 1·00	1·17	1·08, 1·27
December	0·70	0·65, 0·76	0·90	0·82, 0·98

### Fracture rates compared between individuals potentially exposed to vitamin D fortification and non-exposed individuals

Among girls, the rate ratio for the exposed was 1·15 (95 % CI 1·11, 1·20) compared with the non-exposed. Among boys, the rate ratio for the exposed compared with the non-exposed was 1·11 (95 % CI 1·07, 1·14) (Fig. 2 and 5). However, these associations no longer persisted after including the period effects. The downward trend seen in the estimates from the age-cohort model has successfully been assigned as a period effect and there appears to be no systematic variation left in the cohort term based on the residuals. The relative risk for the exposed girls was 1·01 (95 % CI 0·96, 1·05) compared with the non-exposed girls, and the relative risk for exposed boys compared with the non-exposed boys was 1·01 (95 % CI 0·98, 1·04) (Fig. 2 and 5).

For both girls and boys, there was no interaction between season of birth and the exposure to vitamin D fortification in relation to overall fracture risk (girls: *P* 0·23; boys: *P* 0·44).

## Discussion

This study assessed the long-term risk of childhood fractures from the natural experiment of terminating a mandatory margarine fortification programme that until 1 June 1985 fortified all margarine with vitamin D in Denmark. The study did not provide evidence that prenatal exposure to extra vitamin D from fortification was sufficient to influence the risk of fractures in late childhood, regardless of season of birth.

Interestingly, the fortification programme may have taken place against a long-term trend of a decreasing fracture risk, including these particular birth cohorts under study. Thus, we cannot rule out whether the birth cohort results reflect a true adverse effect of vitamin D on bone; one previous study did indeed report borderline significant inverse associations between maternal late pregnancy 25(OH)D concentrations and offspring forearm fractures^(^ ^12^ ^)^. Also, in the Danish National Birth Cohort, mid-pregnancy maternal supplementation with >10 µg vitamin D/d was associated with a 30 % higher risk of offspring forearm fractures^(^ ^37^ ^)^.

Nonetheless, the decreasing trend may have been affected by influences more powerful than the likely impact of vitamin D in the modest amounts of 1·25 µg vitamin D/100 g margarine^(^ ^31^ ^)^. By comparing individuals from entire adjacent birth cohorts that were, or were not, exposed to extra vitamin D from margarine fortification prenatally only, but all unexposed thereafter, we assume that all potential confounders are equally distributed in both groups, and hence that control for confounding is not needed. Though we are unaware of such changes, we cannot exclude the possibility that other societal, environmental or behavioural changes coinciding with the change in fortification practice took place. However, changes in potential risk factors for paediatric fractures during the study period 1996–2012 may also be considered, such as secular trends in weight status, high-impact sports, better safety on playgrounds, onset of puberty, etc. Nonetheless, recent studies have found tendencies for a leveling-off of the trend in overweight and obesity among Danish adolescents (age range 11–16 years) during 2002–2010^(^ ^38^ ^,^ ^39^ ^)^. Similarly, the rate of injuries among children and youth occurring during home and leisure activities (as a proxy for physical activity), that is, at playgrounds and in sports participation, were stable during the period of interest^(^ ^40^ ^)^. An increasing number of Danish children are diagnosed with precocious puberty from the mid-1990s and onwards^(^ ^41^ ^)^, although very few cases in total have been registered in the period 1993–2001 (*n* 670)^(^ ^42^ ^)^. Also, other Danish studies have suggested a decline in the age of onset of puberty, among both girls and boys, from 1991 to 2008^(^ ^43^ ^,^ ^44^ ^)^. However, the decline was about 3–4 months over a 15-year period, and thus considered minor with regard to the adjacent birth cohorts that we studied over a maximum span of 5 years. Hence, the unchanging fluctuations in the trend of these major risk factors of fractures cannot explain the decreasing drift seen in our data.

The decreasing fracture trend may potentially be explained by the marked decrease in bicycle and pedestrian accidents related to road traffic injuries during 1990–2009^(^ ^40^ ^)^, especially as fractures of the shoulders, arms, hands and wrist were those represented in our data set, and are the body parts most likely to be injured in bicycle accidents^(^ ^40^ ^)^. Also, the seasonal pattern in bicycle accidents, with particularly high rates during spring and summer compared with winter and autumn, was similar between the fracture rate pattern in our study and that in other studies^(^ ^45^ ^–^ ^52^ ^)^. Climatically, Denmark has a large seasonal variation in daylight length, temperature and precipitation. This seasonal variation might also contribute to periodic changes in outdoor physical activities among children during the year^(^ ^53^ ^,^ ^54^ ^)^.

The relationship between vitamin D status during fetal life and long-term bone health has not previously been widely examined, and results from earlier observational studies showed either no association or a direct association. In the Avon Longitudinal Study of Parents and Children (ALSPAC) study, UV-B-exposure during the third trimester of pregnancy was used as a proxy for vitamin D status, and the exposure was directly associated with bone mineral content (BMC), bone area and BMD among 6955 children at age 9·9 years^(^ ^8^ ^)^. However, later, another report from the ALSPAC study showed no association with maternal 25(OH)D and total body BMC or spine BMC among 3960 offspring, after adjusting for offspring age by dual-energy X-ray absorptiometry scan^(^ ^10^ ^)^. Correspondingly, Danish Cohort studies showed no association between maternal 25(OH)D concentrations during pregnancy and fractures among the offspring at ages 0–18 and 0–21 years^(^ ^12^ ^,^ ^37^ ^)^. Findings from the Southampton Women Survey suggested that both 25(OH)D and estimated UV-B radiation during late pregnancy (mean gestation week of 34) were directly associated with bone-mineral accrual among 198 children up to age 9 years^(^ ^10^ ^)^. Finally, the Western Australian Pregnancy Cohort (Raine), which followed 341 offspring up to the age of 20 years, showed a direct association between maternal 25(OH)D measured between 16 and 20 weeks of gestation and total body BMC and BMD at age 20 years^(^ ^11^ ^)^. The diverse results from these studies potentially relates to differences in sample sizes, or the covariates adjusted for, and the number and timing of serum 25(OH)D concentration measurement during pregnancy. No previous studies have examined the effect of prenatal food fortification. Intervention studies with supplementation of vitamin D in pregnancy are currently ongoing^(^ ^9^ ^,^ ^55^ ^)^. One of them, the Maternal Vitamin D Osteoporosis Study (MAVIDOS), recently reported that maternal supplementation with 1000IU (25 µg)/d cholecalciferol during pregnancy increased bone mass in winter-born infants only^(^ ^56^ ^)^; however, it could be more than a decade before the first bone fracture outcome data in children are available to properly formulate health policies.

A number of methodological issues deserve attention. From systematic review and meta-analysis of randomised controlled trials, there is evidence that vitamin D-fortified foods generally improve vitamin D status among both children and adults in a dose-dependent manner^(^ ^57^ ^–^ ^59^ ^)^. The Danish margarine fortification programme has been shown to account for approximately 0·36–0·57 µg vitamin D/person per d^(^ ^60^ ^)^. These estimates are based on food disappearance data (food availability for human use), where the average purchasing of margarine was estimated to be 16 000–17 000 g margarine/person per year during the period 1983–1988^(^ ^33^ ^)^, which is equal to 44–46 g margarine/person per d, and the dose-equivalents for vitamin D from fortified margarine is on average 0·55–0·57 µg/person per d^(^ ^33^ ^)^. Furthermore, from the Danish dietary habit survey from 1985, the median vitamin D intake was 2·8 µg/d among women aged 23–50 years^(^ ^61^ ^)^. Margarine consumption contributed to 13 % of this vitamin D intake, which is equivalent to 0·36 µg^(^ ^61^ ^)^. It is possible that this amount was too small to influence fracture risk at the population level, even when we confined the analysis to those pregnancies with habitually low vitamin D during late trimesters, that is, those giving birth during winter months. Unfortunately, we lack information on vitamin D status among Danish women in the reproductive age during the fortification period, but for comparision, a study showed that among 850 pregnant women recruited from the second-largest city in Denmark in 1988–1989 (the period without fortification), only 6·3 of the pregnant women had a serum 25(OH)D concentration ≤25 nmol/l^(^ ^12^ ^)^; the median concentrations of 76·2 (95 % CI 23·0, 152·1) nmol/l were also above the ‘optimal level’ as stated by the US Endocrine Society^(^ ^62^ ^,^ ^63^ ^)^. For replication, the Finish vitamin D fortification programme (initiated in 2003) may be an option because Finland, like Denmark, has access to nationwide individual registration of fractures^(^ ^64^ ^)^.

There are also limitations to the statistical model. The age-period analysis assumed that the overall trend in the fractures rates could be attributed to a period effect. Bearing in mind the identifiability problem between age, period and cohort, the data cannot inform whether the age-period approach is more appropriate than the age-cohort analysis. To help elucidate the ‘true’ period effect, we suggest, at this point, a National trend analysis of paediatric fractures for birth cohorts from several decades and not only births from 1983 to 1988.

The strength of the present study lies with the use of comprehensive registers covering the entire Danish population. This enables us to capture all fracture information in the study population from the NPR, which is a high-quality national mandatory registration system initiated in 1977. The accuracy of the NPR has not been formally assessed as regards paediatric bone ICD coding. Fracture diagnosis coding has high precision in adults^(^ ^65^ ^)^, and treatment of childhood fractures takes place at the same hospital units as treatment of fractures in adulthood, with very little extent of fracture treatment in general practice.

### Conclusion

The study did not provide evidence that prenatal exposure to extra vitamin D from a mandatory fortification programme, adding1·25 µg vitamin D/100 g margarine, was sufficient to influence the risk of fractures in late childhood, regardless of season of birth. Replication studies are needed. There was a decreasing trend in fracture events occurring in the birth cohort of 1983–1988, which might be explained by secular trends of bicycle accidents in the period, rather than by differences in the birth cohorts.

## Supplementary material

For supplementary material accompanying this paper visit http://dx.doi.org/10.1017/S000711451700071X. click here to view supplementary material
