# Supplementary material for: Prenatal exposure to vitamin D from fortified margarine and risk of fractures in late childhood: period and cohort results from 222 000 subjects in the D-tect observational study
Source: Br J Nutr. 2017 Apr 10;117(6):872–81. doi: 10.1017/S000711451700071X (PMC5426325; doi:10.1017/S000711451700071X)
Supplement: Supplementary file 1 [file S000711451700071Xsup001.zip › S000711451700071Xsup002.pdf]

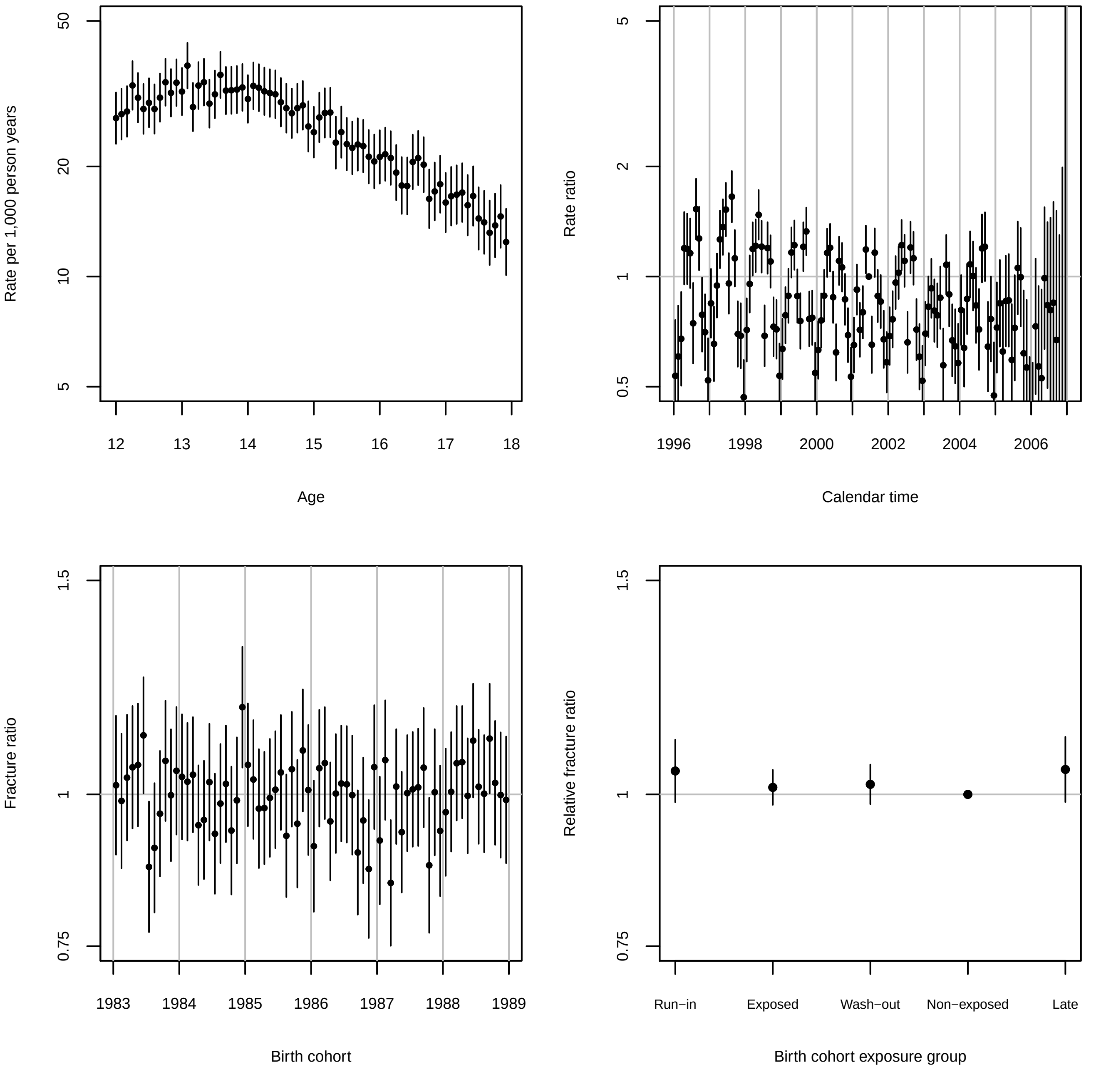
Rate per 1,000 person years
50
20
10
5
12
13
14
15
16
17
18
Age
Rate ratio
5
2
1
0.5
1996
1998
2000
2002
2004
2006
Calendar time
Fracture ratio
1.5
1
0.75
1983
1984
1985
1986
1987
1988
1989
Birth cohort
Relative fracture ratio
1.5
1
0.75
Run−in
Exposed
Wash−out
Non−exposed
Late
Birth cohort exposure group
