# Supplementary material for: Prenatal exposure to vitamin D from fortified margarine and risk of fractures in late childhood: period and cohort results from 222 000 subjects in the D-tect observational study
Source: Br J Nutr. 2017 Apr 10;117(6):872–81. doi: 10.1017/S000711451700071X (PMC5426325; doi:10.1017/S000711451700071X)
Supplement: Supplementary file 1 [file S000711451700071Xsup001.zip › S000711451700071Xsup005.docx]

Figure 0, boys 12-18 y. Age and birth cohort effects for boys born in 1983-1988. Upper left: Age specific fracture rates pr. 1000 person years and 95% CI for boys born in September 1986. Upper right: Rate ratio relative to September 1986 cohort. Lower left: Rate ratios by birth cohort exposure groups (“Non-exposed” cohort is reference). Lower right: Rate ratios by birth cohort exposure group and season of birth (Birth season “August-October” and “Non-exposed” cohort is reference).

Figure 0, boys 12-18 y. Age and period effects for boys with fractures occurring from 1996-2007. Upper left: Age specific fracture rates pr. 1000 person years and 95% CI for boys in June 2001. Upper right: Rate relative to the July 2001 rate. Lower left: Observed vs. expected number of fractures conditional on the estimated age and period rates. Lower right: Cohort effect by birth cohort exposure group relative to the cohort effect in the “Non-exposed” cohort.

Figure 0, girls 12-18 y. Age and birth cohort effects girls born in 1983-1988. Upper left: Age specific fracture rates pr. 1000 person years and 95% CI for girls born in September 1986. Upper right: Rate ratio relative to September 1986 cohort. Lower left: Rate ratios by birth cohort exposure groups ( “Non-exposed” cohort is reference). Lower right: Rate ratios by birth cohort exposure group and season of birth (Birth season “August-October” and “Non-exposed” cohort is reference).

Figure 0, girls 12-18 y. Age and period effects for girls with fractures occurring from 1996-2007. Upper left: Age specific fracture rates pr. 1000 person years and 95% CI for girls in June 2001. Upper right: Rate relative to the July 2001 rate. Lower left: Observed vs. expected number of fractures conditional on the estimated age and period rates. Lower right: Cohort effect by birth cohort exposure group relative to the cohort effect in the “Non-exposed” cohort.
