# Supplementary material for: Prenatal exposure to vitamin D from fortified margarine and risk of fractures in late childhood: period and cohort results from 222 000 subjects in the D-tect observational study
Source: Br J Nutr. 2017 Apr 10;117(6):872–81. doi: 10.1017/S000711451700071X (PMC5426325; doi:10.1017/S000711451700071X)

Supplementary Figure 1.

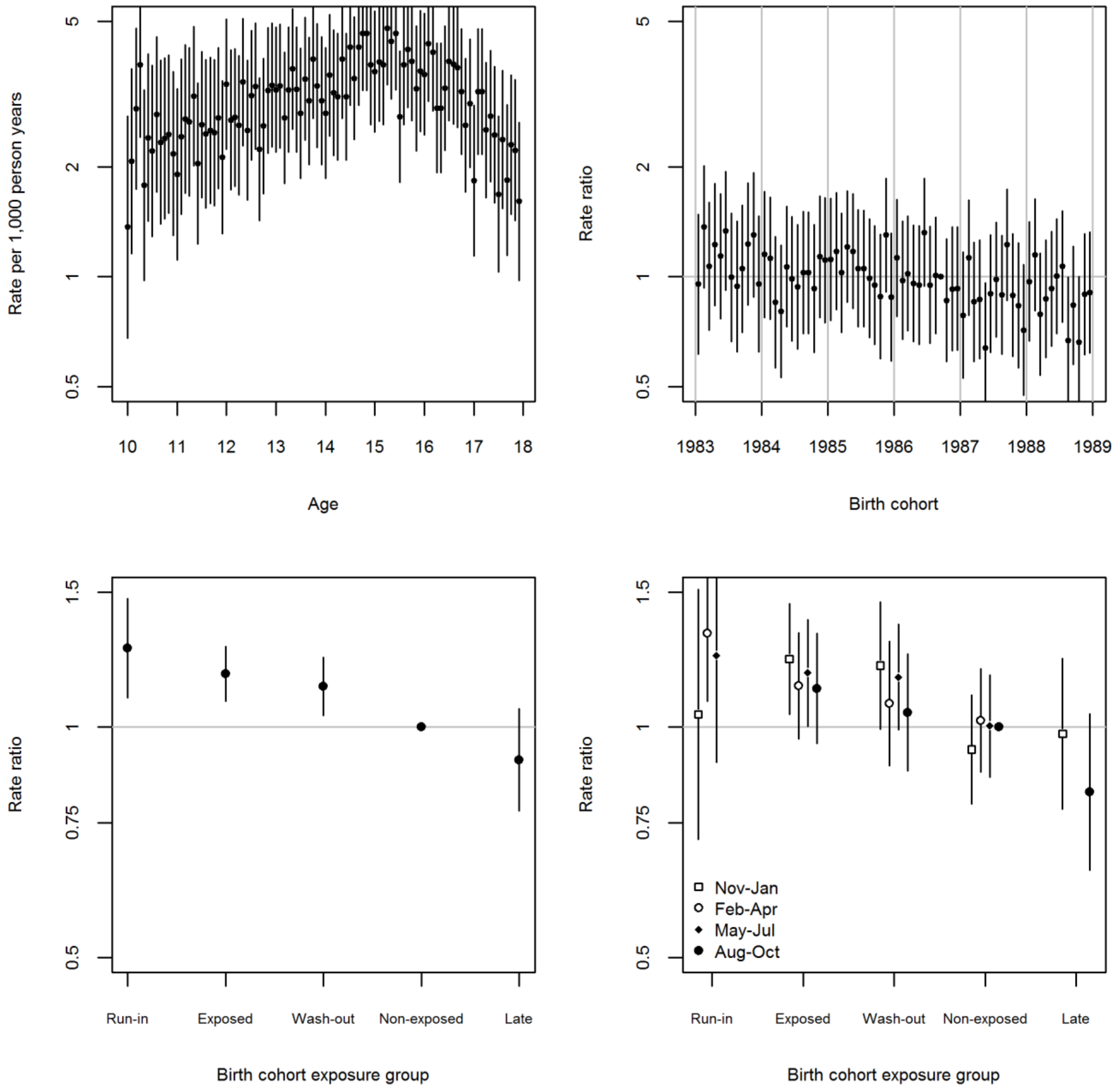

Supplementary Figure 2.

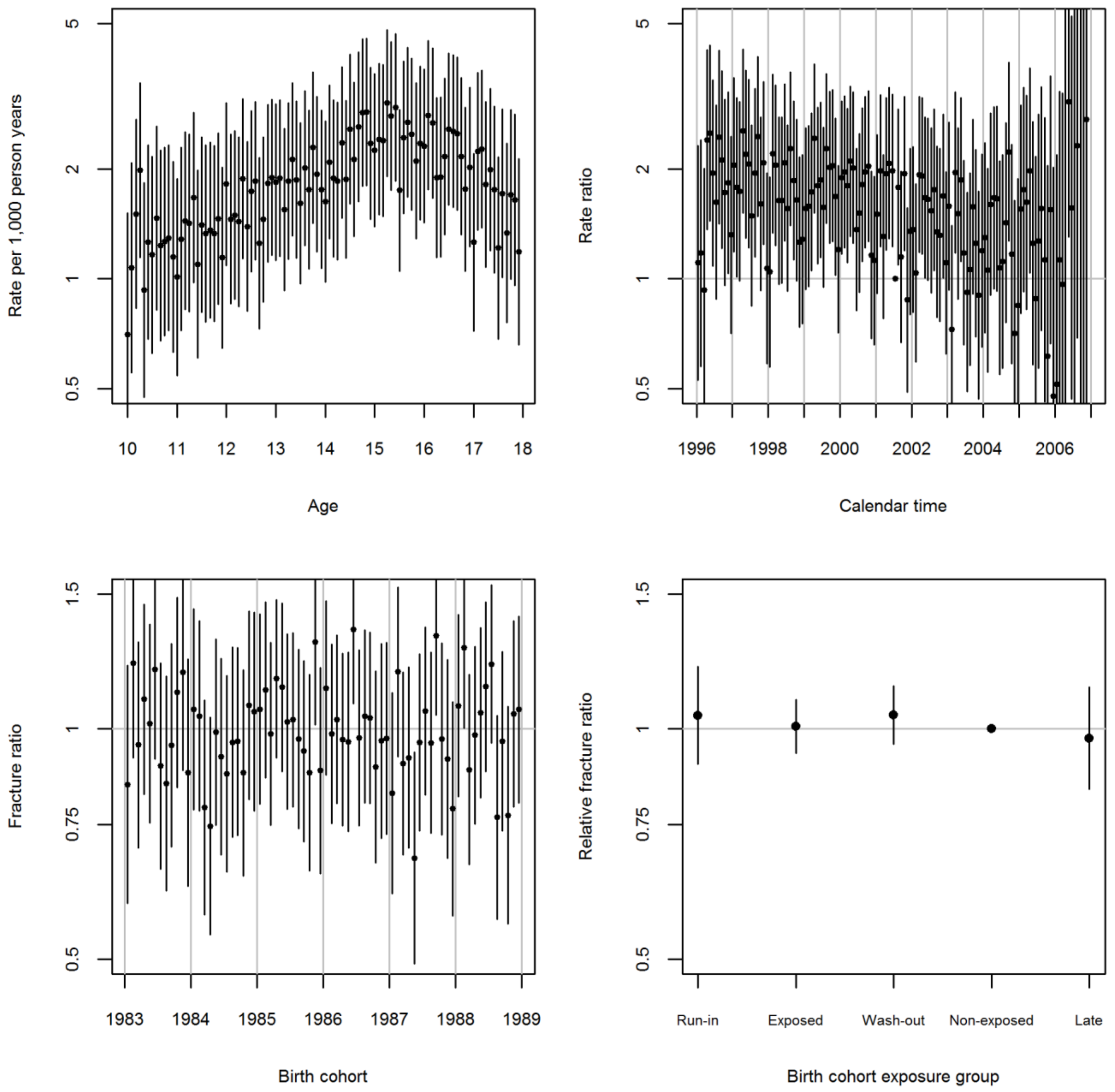

Supplementary Figure 3.

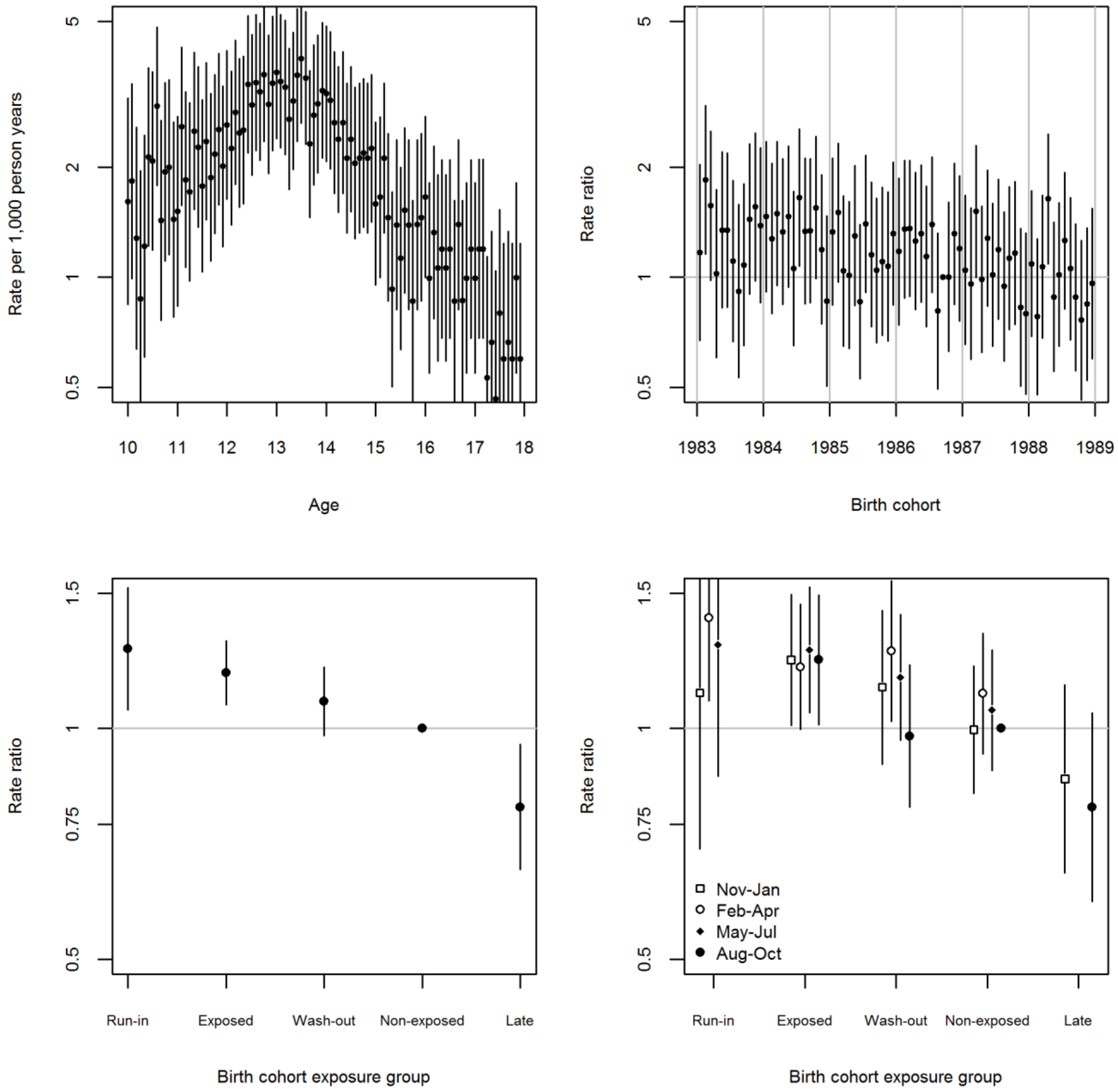

Supplementary Figure 4.

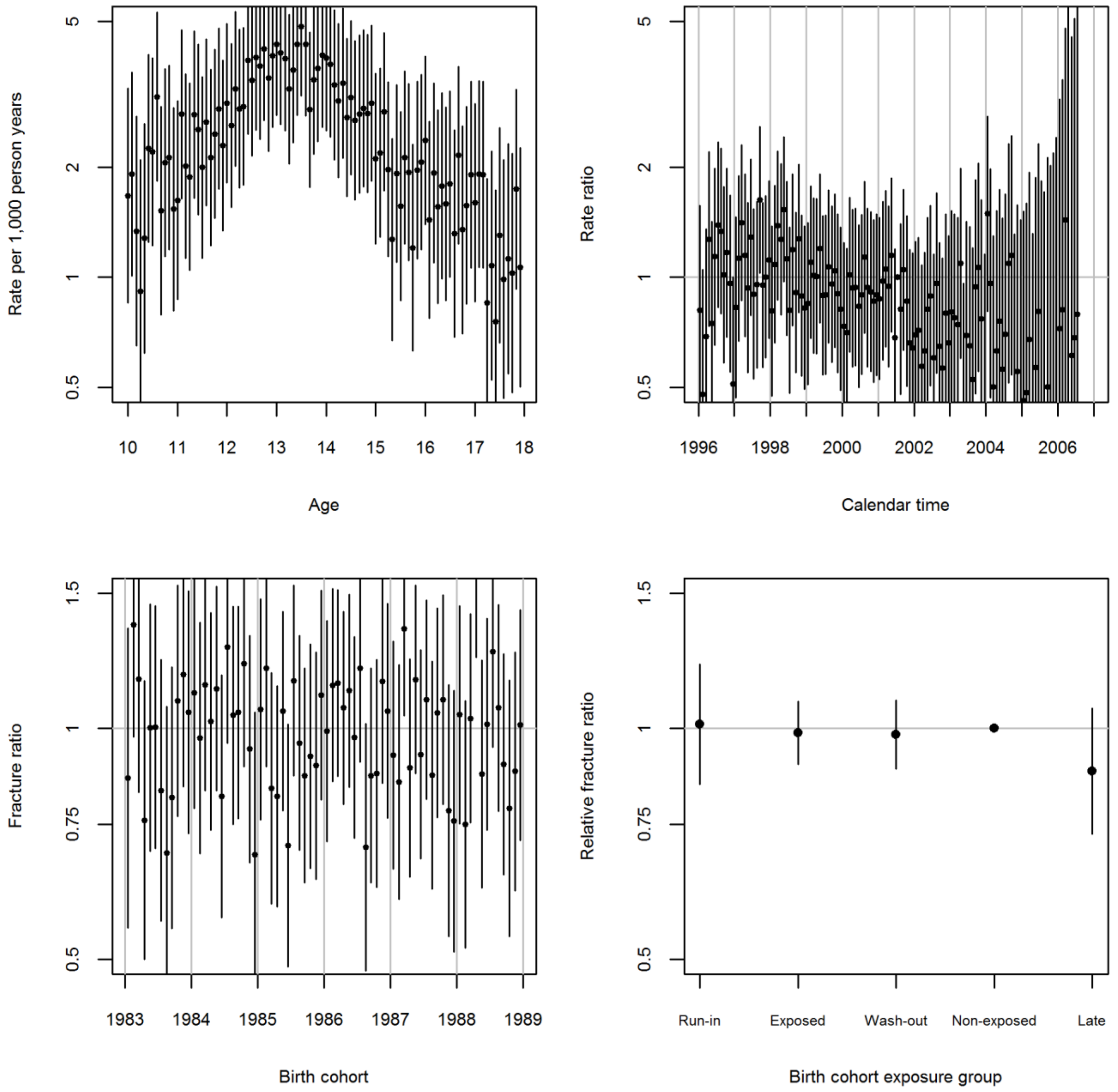

Supplementary Figure 5.

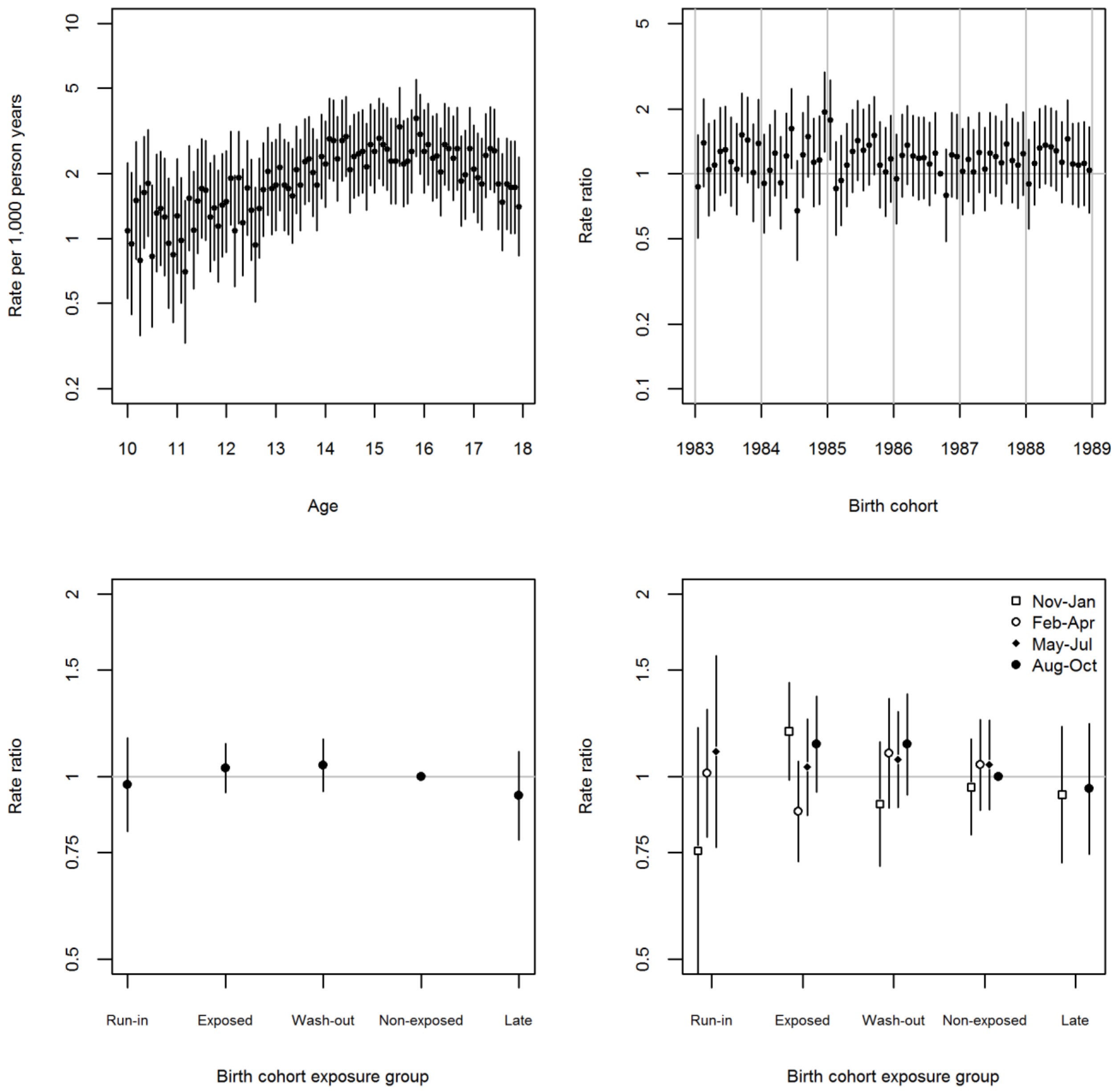

Supplementary Figure 6.

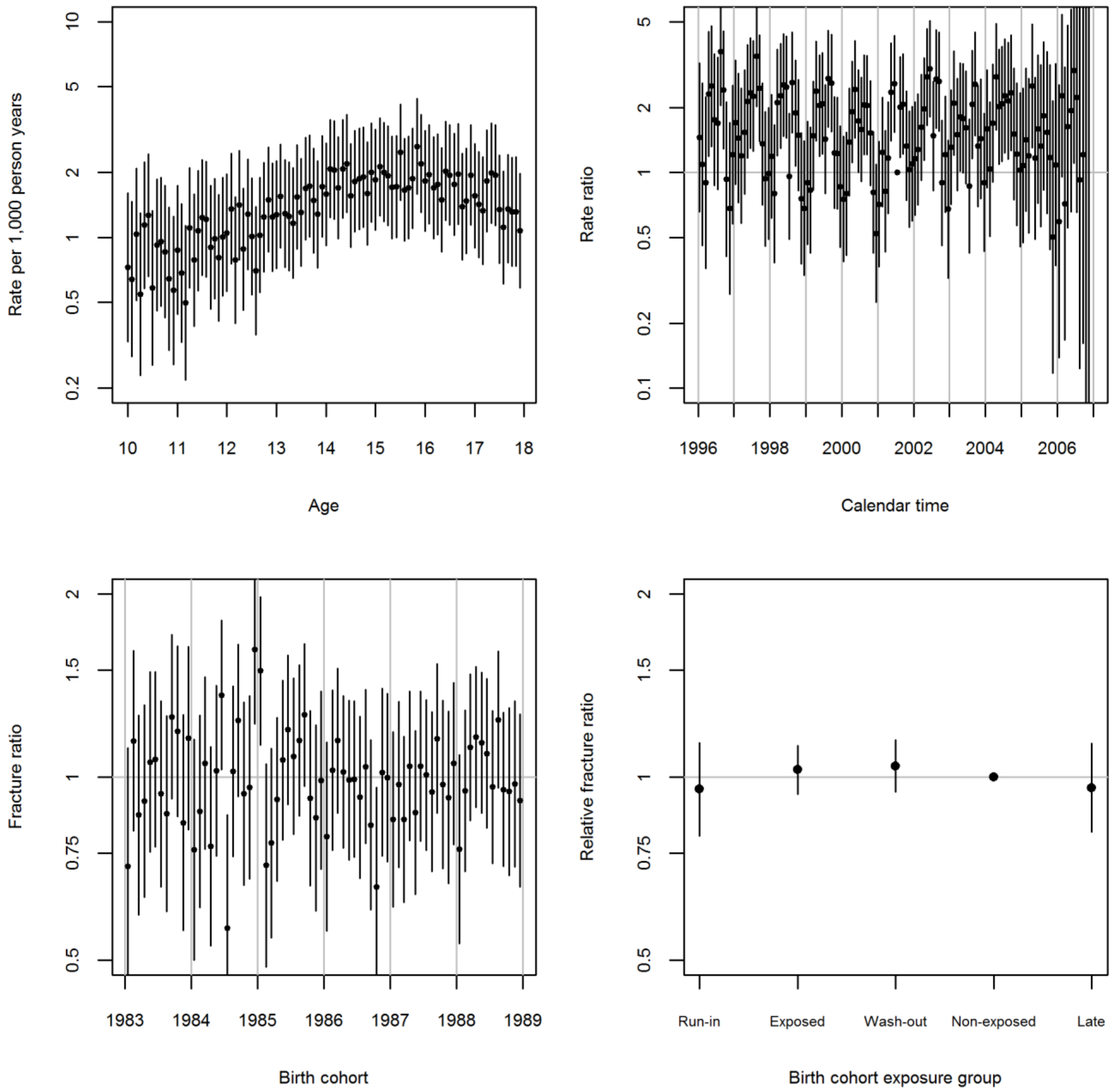

Supplementary Figure 7.

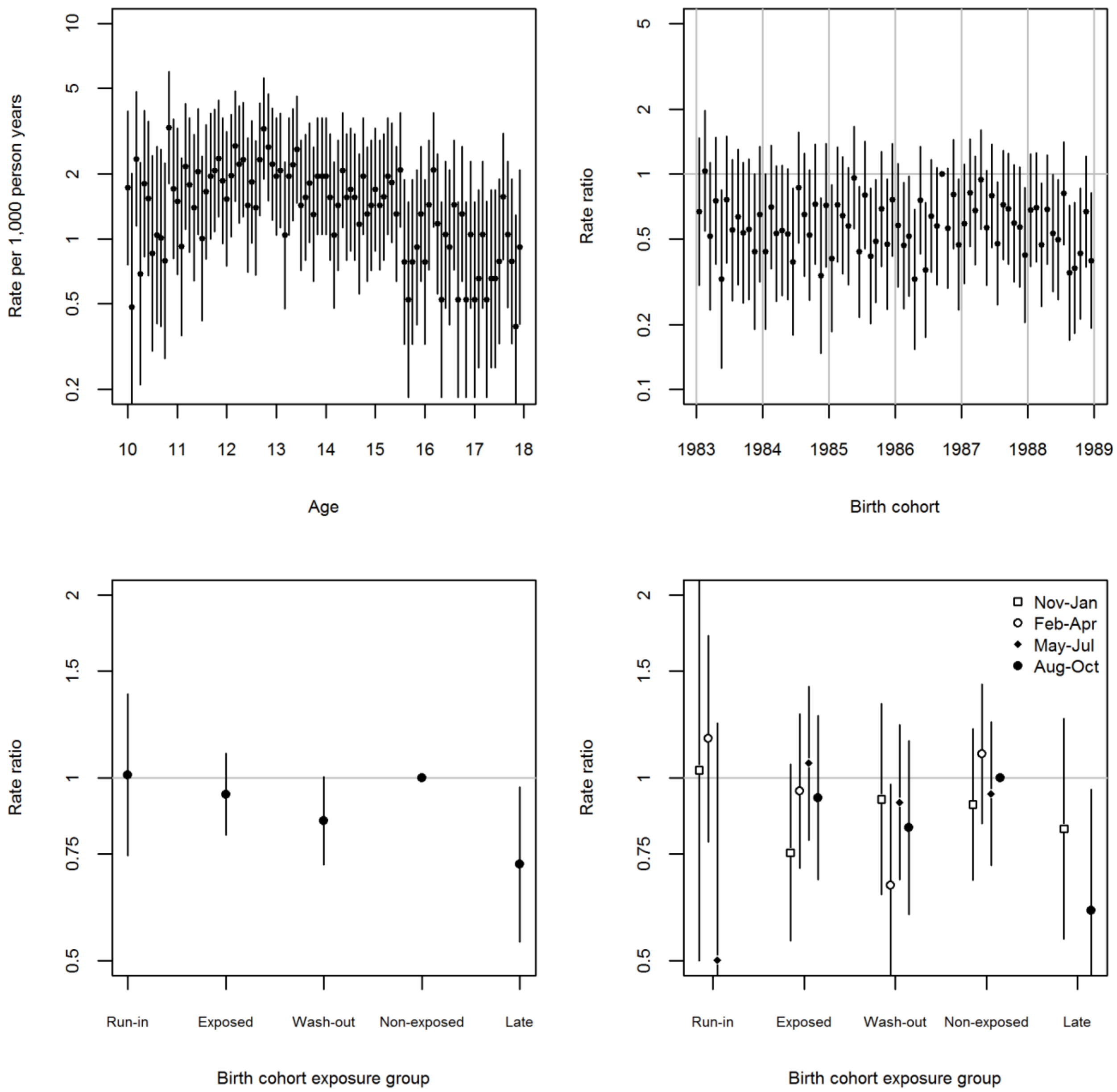

Supplementary Figure 8.

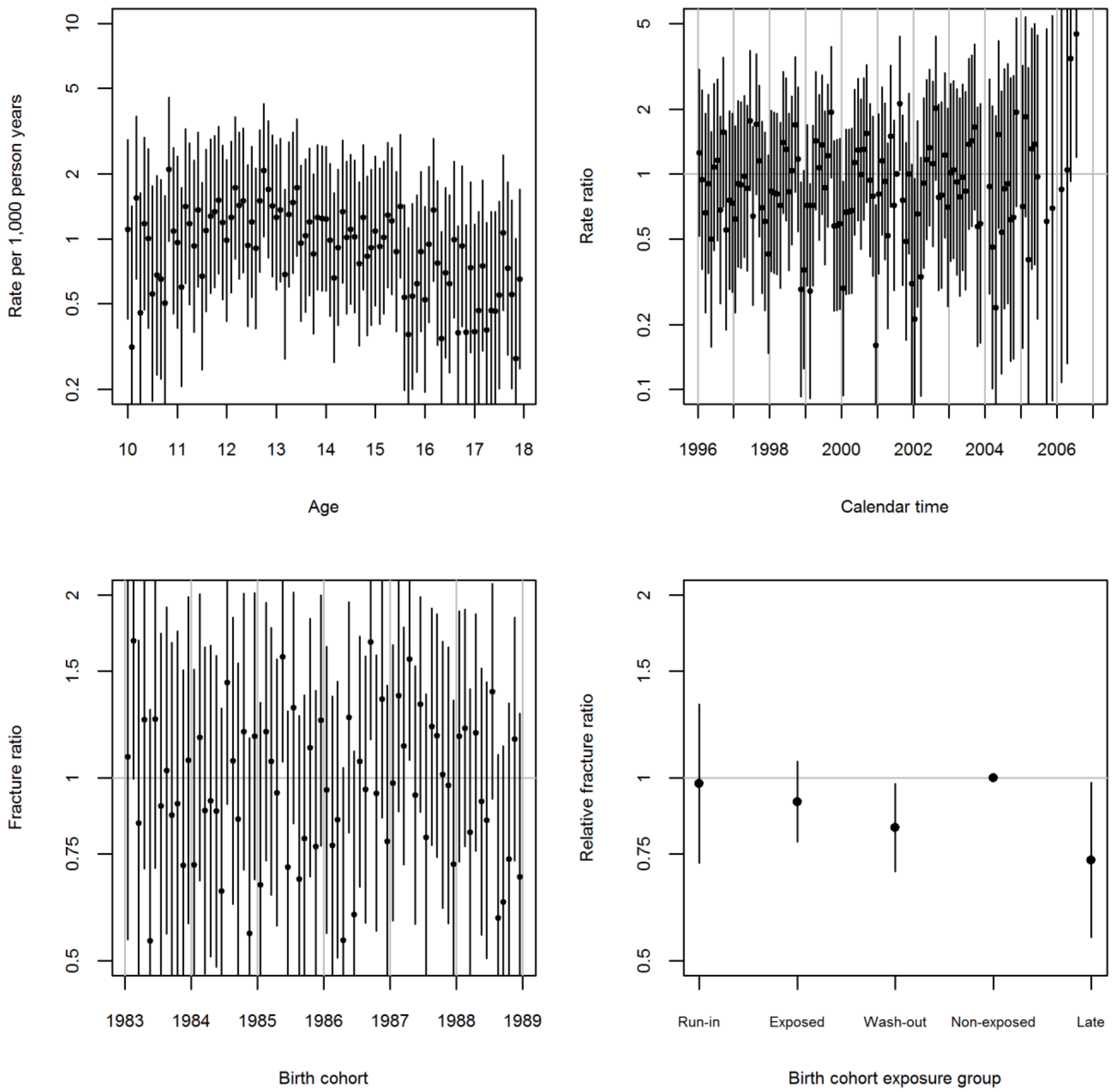

Supplementary Figure 9.

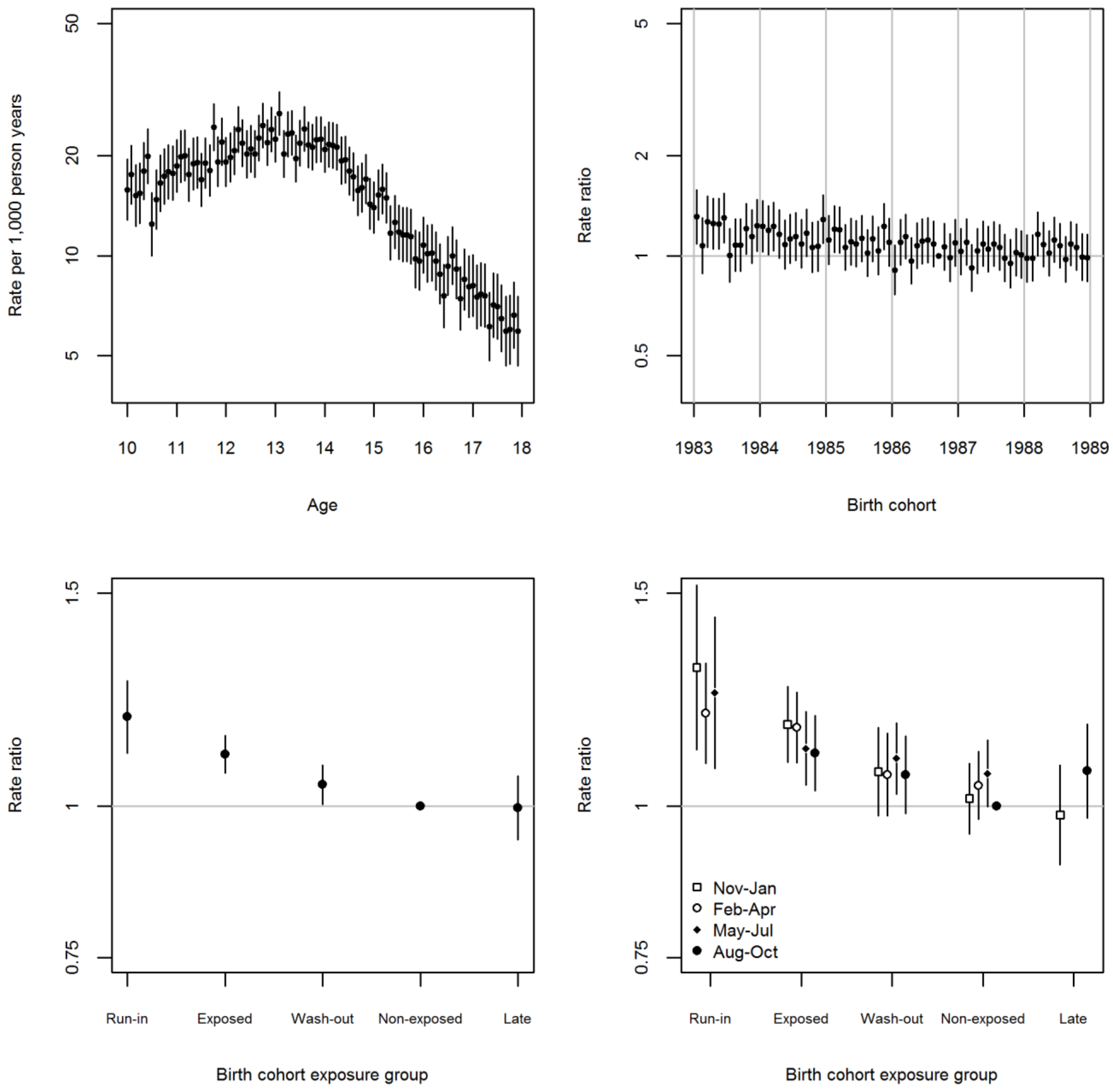

Supplementary Figure 10.

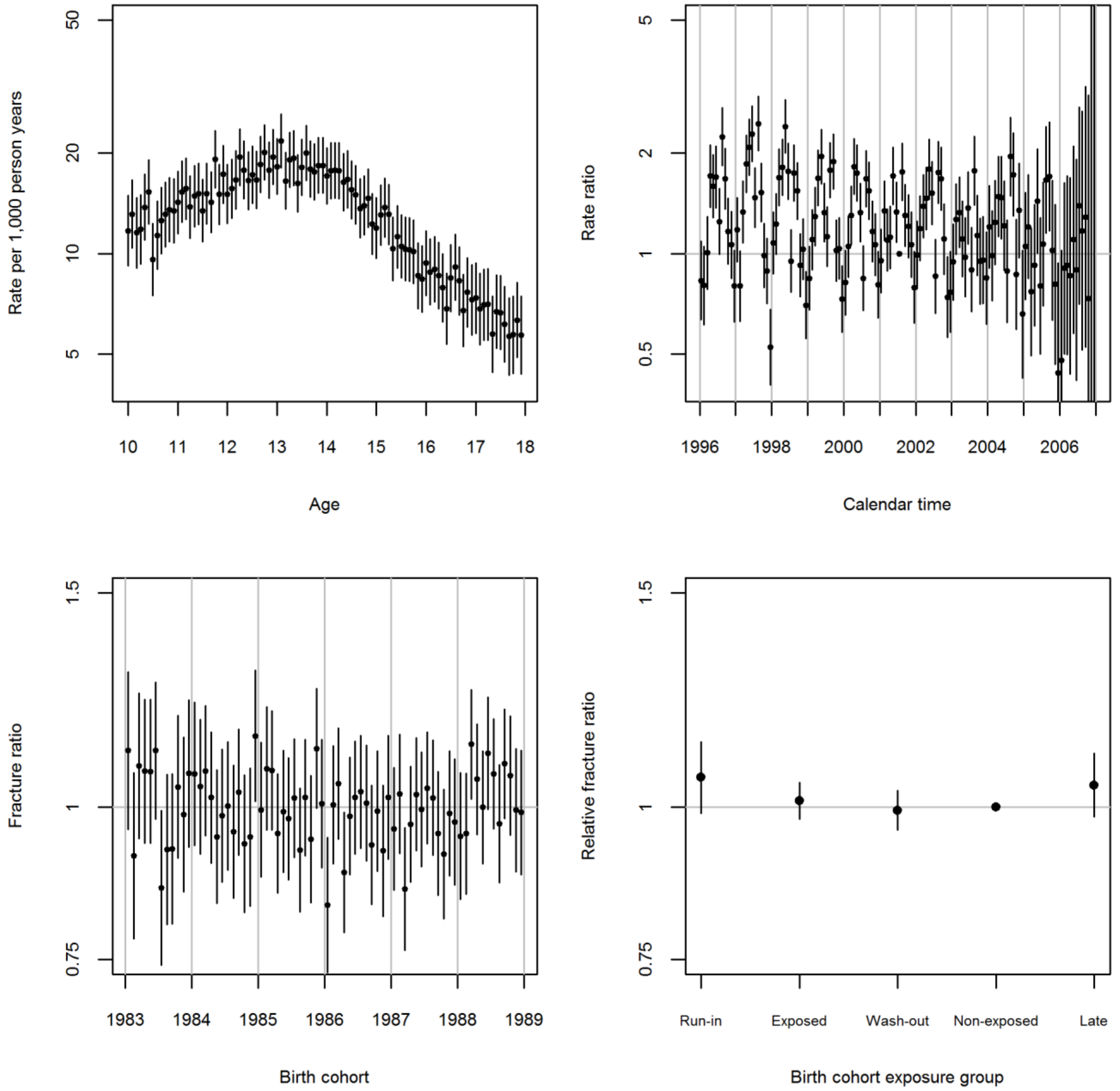

Supplementary Figure 11.

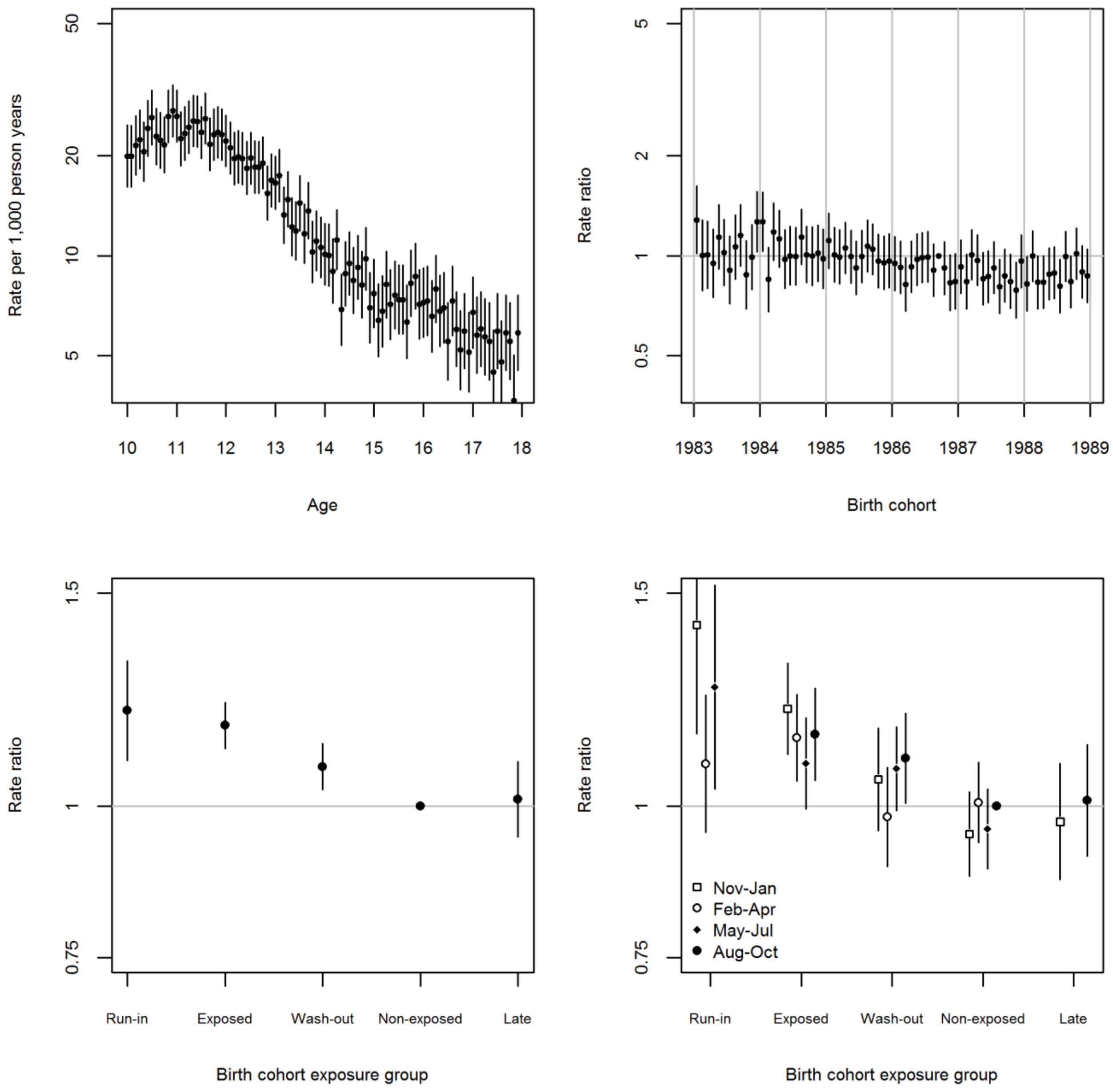

Supplementary Figure 12.

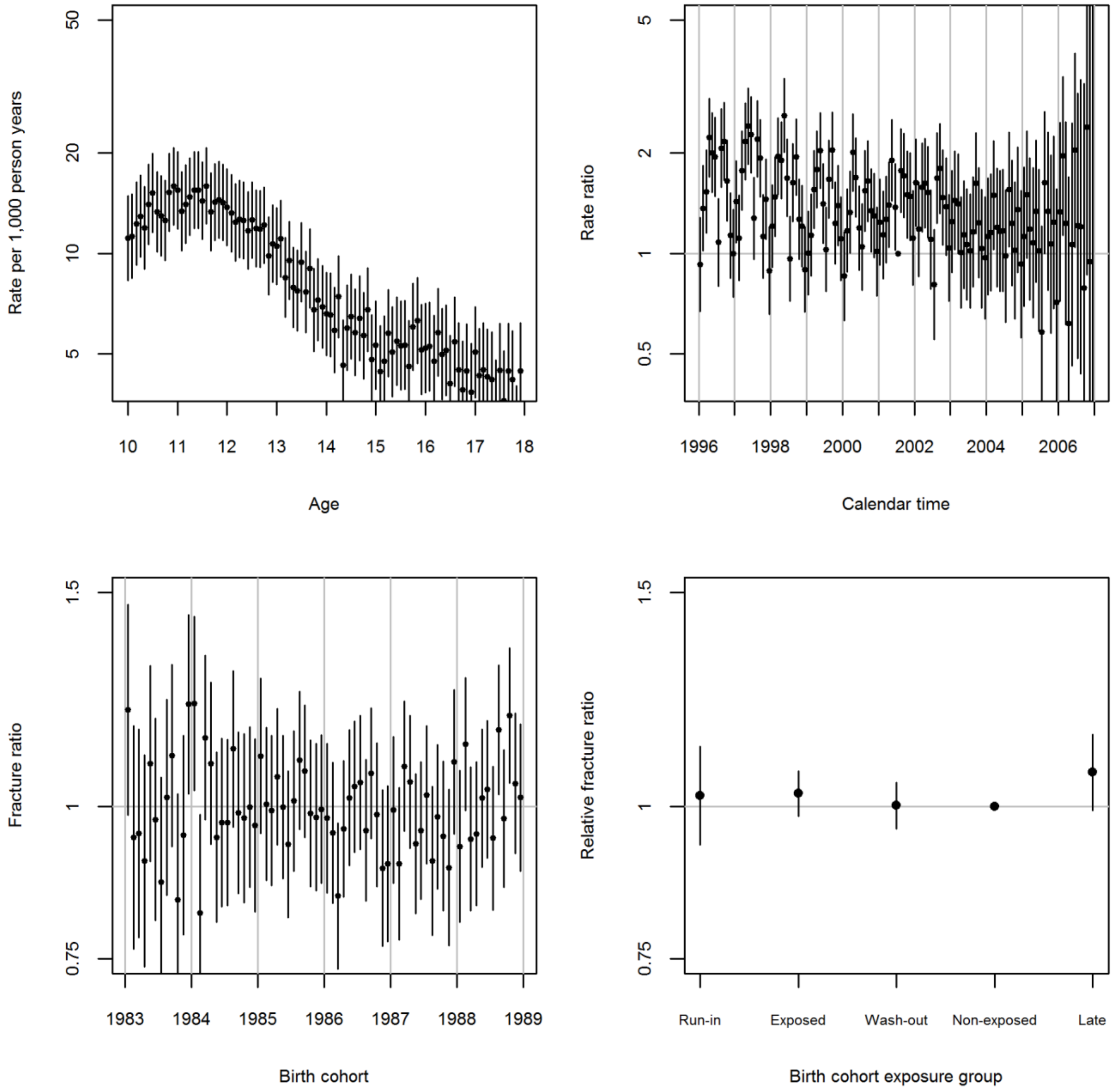

Supplement: Supplementary file 1 [file S000711451700071Xsup001.zip › S000711451700071Xsup006.pdf]
