# Supplementary figures and images for: Prenatal exposure to vitamin D from fortified margarine and risk of fractures in late childhood: period and cohort results from 222 000 subjects in the D-tect observational study
Source: Br J Nutr. 2017 Apr 10;117(6):872–81. doi: 10.1017/S000711451700071X (PMC5426325; doi:10.1017/S000711451700071X)

Rate per 1,000 person years

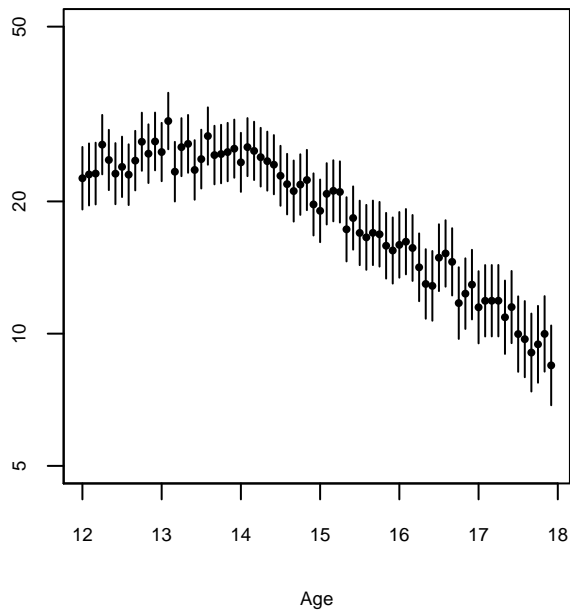

Rate ratio

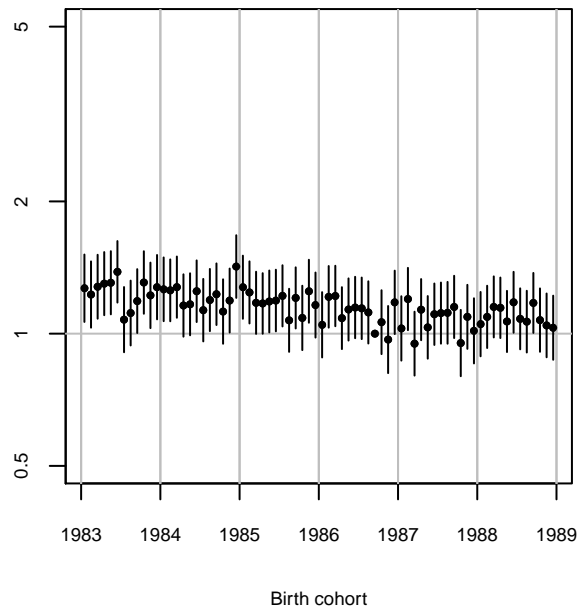

Rate ratio

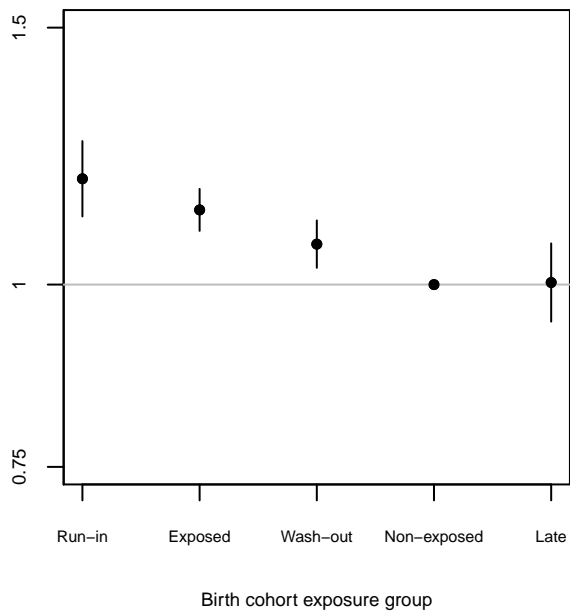

Rate ratio

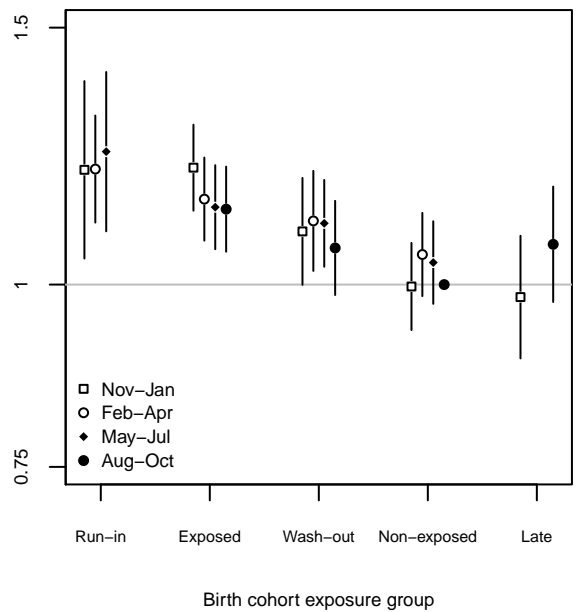

Supplement: Supplementary file 1 [file S000711451700071Xsup001.zip › S000711451700071Xsup001.pdf]
